# Legacy or colonization? Posteruption establishment of peregrine falcons (*Falco peregrinus*) on a volcanically active subarctic island

**DOI:** 10.1002/ece3.2631

**Published:** 2016-12-12

**Authors:** Sarah A. Sonsthagen, Jeffrey C. Williams, Gary S. Drew, Clayton M. White, George K. Sage, Sandra L. Talbot

**Affiliations:** ^1^ US Geological SurveyAlaska Science CenterAnchorageAKUSA; ^2^ US Fish and Wildlife ServiceAlaska Maritime National Wildlife RefugeHomerAKUSA; ^3^ Department of Plant and Wildlife Sciences and Monte L. Bean Life Science MuseumBrigham Young UniversityProvoUTUSA

**Keywords:** colonization, dispersal, *Falco peregrinus*, genetic legacy, Kasatochi Island, peregrine falcon

## Abstract

How populations and communities reassemble following disturbances are affected by a number of factors, with the arrival order of founding populations often having a profound influence on later populations and community structure. Kasatochi Island is a small volcano located in the central Aleutian archipelago that erupted violently August 8, 2008, sterilizing the island of avian biodiversity. Prior to the eruption, Kasatochi was the center of abundance for breeding seabirds in the central Aleutian Islands and supported several breeding pairs of peregrine falcons (*Falco peregrinus*). We examined the reestablishment of peregrine falcons on Kasatochi by evaluating the genetic relatedness among legacy samples collected in 2006 to those collected posteruption and to other falcons breeding along the archipelago. No genotypes found in posteruption samples were identical to genotypes collected from pre‐eruption samples. However, genetic analyses suggest that individuals closely related to peregrine falcons occupying pre‐eruption Kasatochi returned following the eruption and successfully fledged young; thus, a genetic legacy of pre‐eruption falcons was present on posteruption Kasatochi Island. We hypothesize that the rapid reestablishment of peregrine falcons on Kasatochi was likely facilitated by behavioral characteristics of peregrine falcons breeding in the Aleutian Islands, such as year‐round residency and breeding site fidelity, the presence of an abundant food source (seabirds), and limited vegetation requirements by seabirds and falcons.

## 1 Introduction

How populations are founded and how communities reassemble following disturbances, such as volcanic eruptions, are affected by a number of factors, including the severity of disturbance, priority effects (dispersal and arrival order; Hoverman & Relyea, 2008), and availability of propagules (Mazzola et al., 2011) either from survivors (representing legacy biodiversity, Walker et al., 2013), or from colonizers (representing founder biodiversity). Most studies have examined these factors retrospectively (Fleischer, McIntosh, & Tarr, 1998; Percy et al., 2008; Ricklefs, 2010; Shaw, 1996; Yang, Bishop, & Webster, 2008), as opportunities to study community reassembly following major disturbances are rare. As colonization of newly sterilized areas can occur rapidly (e.g., plants on Krakatau, Thornton, 1984; birds on Surtsey, Petersen, 2009; predaceous flies on Kasatochi, Walker et al., 2013), catastrophic disturbance events that simplify community relationships via the elimination of most or all flora and fauna provide particularly useful opportunities to study deterministic versus stochastic processes influencing the reassembly of communities (Mazzola et al., 2011; Walker et al., 2013).

Initial founding events and the expansion of founding individuals across the landscape may profoundly influence later populations (Yang et al., 2008) and community structure in newly created habitats. For example, among closely related species in the Hawaiian Archipelago, there is generally a linear relationship between island age and genetic distance (sequential radiation), suggestive of rapid colonization following island genesis, yet evidence of subsequent colonization was not observed (e.g., Fleischer et al., 1998; Percy et al., 2008; Shaw, 1996). This pattern of colonization illustrates the importance of initial founding events, as initial founders may impact the ability of subsequent dispersers to successfully colonize (Waters, Fraser, & Hewitt, 2012). Therefore, species and/or individuals that survived the eruption may play a pivotal role in the success of subsequent colonization attempts by other species or individuals (Franklin, 2005), ultimately impacting community assemblage posteruption (Walker et al., 2013).

Establishment, or reestablishment, of terrestrial fauna and associated food webs on islands following major disturbances (or new geological formation) can also depend upon the presence of plants for nesting substrate (i.e., birds on Anak Krakatau; Thornton, Zann, & Stephenson, 1990) and associated food base (i.e., prey for insectivores/carnivores or vegetation for herbivores, frugivores, and nectarivores; Walker et al., 2013). Thus, establishment of local breeding populations for certain species lags until a sufficient level of habitat development has occurred. This limitation, however, may favor rapid colonization and establishment of species that are not dependent upon terrestrial vegetation, such as seabirds and marine mammals. Highly mobile animal species that rely on the marine environment for their food base should not be constrained in their ability to rapidly colonize or recolonize disturbed islands. The limited habitat requirements of seabirds are exemplified on Surtsey Island, which emerged off the south coast of Iceland in an extended series of eruptions. Only 2 weeks after the eruption began, gulls (*Larus* sp.) were observed landing on the island between eruption events (Gudmundsson, 1966; Petersen, 2009). Within 7 years of emergence of the island, 3 years after the cessation of volcanic activity, marine birds started nesting on the island (Fridriksson & Magnússon, 1992; Petersen, 2009). Similarly, marine birds increased in abundance following the eruption on San Benedicto Island, Islas Revillagigedo, Mexico, despite poor survival of seeds and vegetation (Ball and Gluscksman 1975). Therefore, highly vagile species characterized by minimal terrestrial habitat requirements should be early founders in newly sterilized areas and play a pivotal role in how communities are reassembled, through, for example, the facilitation of subsequent colonization of other species via passive dispersal, or provision of a food source.

**Figure 1:**
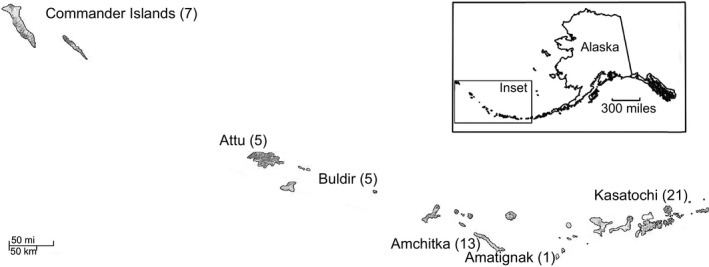
Localities of peregrine falcon populations sampled in the Aleutian archipelago with sample sizes in parentheses

**Figure 2:**
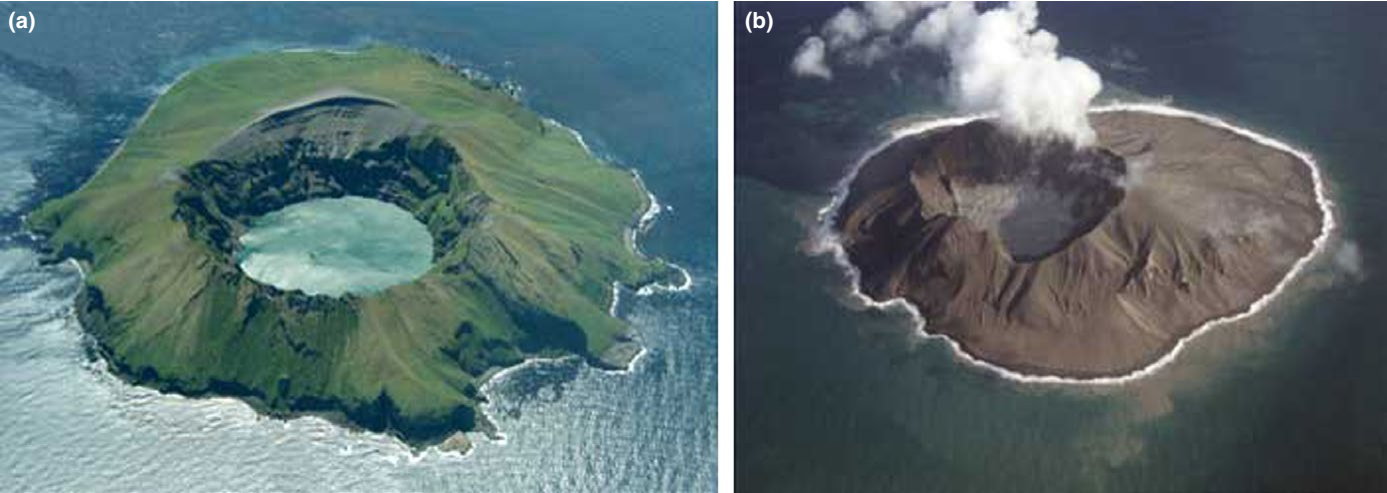
Photographs of Kasatochi Island (a) pre‐eruption, July 2008, and (b) posteruption, October 2008. 
Photographic credit: Jerry Morris (pilot), Security Aviation

Kasatochi Island, part of the U. S. Fish and Wildlife Service Alaska Maritime National Wildlife Refuge (AMNWR), is a small volcano (7.5 km^2^) located in the Andreanof island group in the central Aleutian archipelago, 19 km from the nearest land mass (Figure 1). The violent eruption of the Kasatochi volcano on August 8, 2008 completely covered the island, as well as near‐shore intertidal and shallow subtidal areas, with thick volcanic deposits that devastated wildlife nesting and foraging habitat (Figure 2; DeGange, Byrd, Walker, & Waythomas, 2010). Biological resources on Kasatochi Island had been monitored annually by AMNWR staff from 1996 to 2008, given the island's importance as a center of abundance for breeding seabirds in the central Aleutian Islands (Drummond & Larned, 2007). In particular, least auklets (*Aethia pusilla*) and crested auklets (*A. cristatella*) nested in immense numbers (>200,000 individuals; Williams, Drummond, & Buxton, 2010). By the date of the eruption in 2008, many seabirds had finished breeding for the year, and most adults had left the island; it was assumed they survived to return the following year (Williams et al., 2010). Several other seabird species were still breeding and along with nonfledged young were entombed or perished otherwise in the eruption.

**Figure 3:**
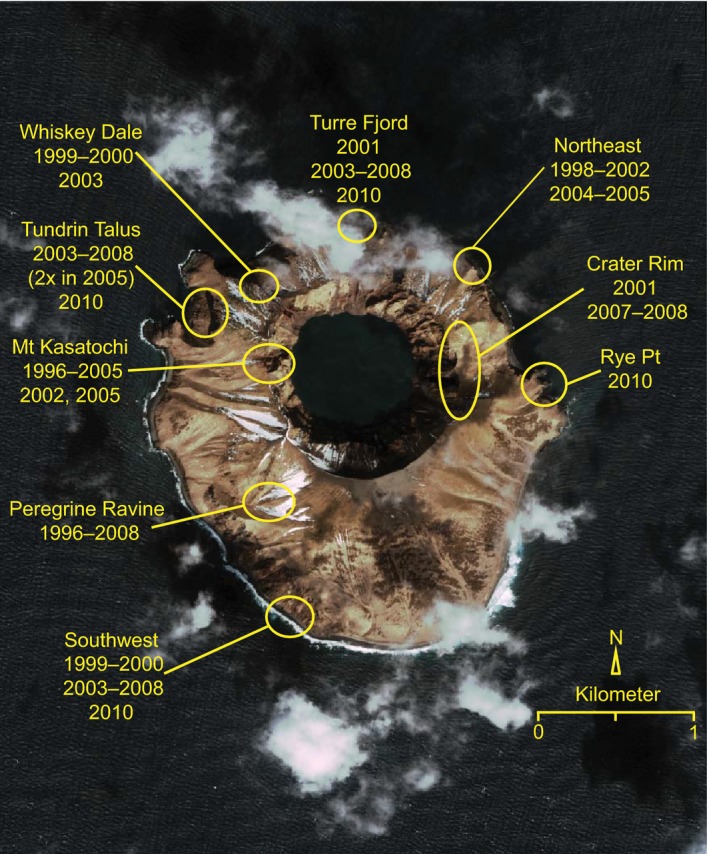
Localities of historical (1996–2008) and current (2010) eyries for peregrine falcons on Kasatochi Island (52.17°N, 175.51°W)

Similar to other islands along the Aleutian chain that host large seabird colonies, a relatively large number of peregrine falcons (*Falco peregrinus*) nested on Kasatochi (e.g., 9 eyries total, 2–6 active eyries in a given year pre‐eruption, along ca. 10 km of coastline [Figure 3] versus an average density of one pair every 10–16 km of coastline in the Aleutian Island Rat Island Group; White, 1976). Unlike seabirds, peregrine falcons and the other primary avian predator in the Aleutian Islands, bald eagles (*Haliaeetus leucocephalus*), are nonmigratory and remain close to their breeding islands throughout the year (White, 1975; White, Emison, & Williamson, 1971). Given this general breeding site fidelity, it was unclear whether avian predators and their fledglings survived the eruption. However, the presence of peregrine falcons (almost exclusively predatory) and bald eagles (partial scavengers) within the first year or two, respectively, following the eruption suggested that these species quickly recolonized, or at least utilized resources on, posteruption Kasatochi. It is not known, however, whether individual birds that occupied pre‐eruption Kasatochi returned posteruption (e.g., represented legacy biodiversity), or whether they were new colonizers. Prior investigation of population‐ and regional‐level genetic structuring suggests peregrine falcons occupying the Aleutian Islands show significant regional‐level structuring and are genetically distinguishable from peregrine falcons occupying habitats elsewhere in Alaska, but show lower levels of structuring across island groups (S. L. Talbot, unpublished data). Thus, levels of natal site fidelity (philopatry) are apparently not sufficiently high to isolate specific island populations, suggesting that individual peregrines occupying posteruption Kasatochi cannot necessarily be assumed to represent legacy biodiversity.

**Figure 4:**
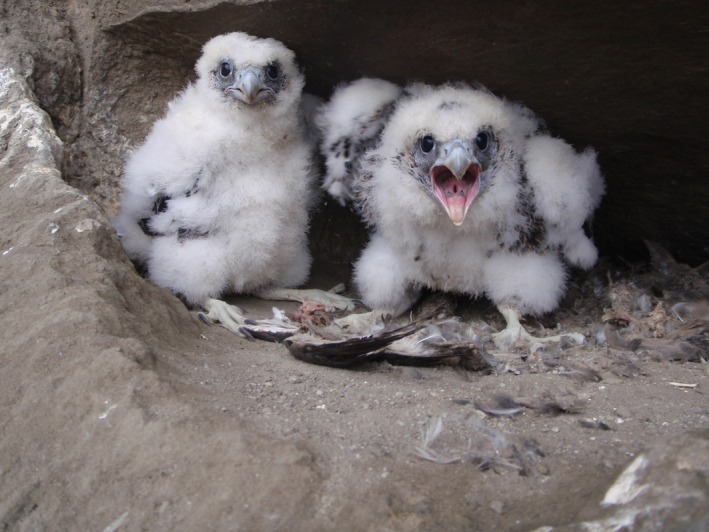
Two peregrine falcon young that were the first known successful avian breeding attempt on posteruption Kasatochi Island (June 2010; photograph by Jeffrey Williams)

Testing for relative importance of in situ and ex situ survival vs. colonization following disturbances, such as volcanic eruptions, is often limited due to lack of historical information about predisturbance residents (Walker et al., 2013). This limitation can be overcome if historical data are sufficient to distinguish colonizers from survivors (Yang et al., 2008). Here, we examine the reestablishment of the peregrine falcon on Kasatochi Island following the 2008 eruption, using genetic data obtained from feather samples collected pre‐ and posteruption and eggshell membranes collected posteruption. Prior to the eruption, the nine eyries used by peregrine falcons were known to be active for three to 11 years from 1996 to 2008 (Figure 3; J. C. Williams, unpublished data). In the first year following the eruption, peregrine falcons were present on the island, although no breeding attempts were observed. In 2010, 2 years posteruption, one eyrie located on the east side of the island at Rye Point (Figure 3) was confirmed as active. Peregrines nesting at this eyrie fledged two young, in the first known successful avian breeding attempt on posteruption Kasatochi (Figure 4). It is possible that at least one other eyrie was established in 2010, but visual confirmation was not possible (J. C. Williams, unpublished data). Our study addressed two questions: (1) Are the peregrines nesting on Kasatochi Island posteruption the same individuals that nested pre‐eruption; and (2) If not, what is the genetic relationship among peregrines pre‐ and posteruption?

## 2 Methods

### 2.1 Samples

Molted feathers (total *n *=* *12; four adults and eight juveniles) found on beaches or near eyries and eggshell membranes (*n *=* *2; 2010) were collected at peregrine falcon eyries posteruption (2009–2011) of Kasatochi Island. It should be noted that juvenile feathers were molted from adult (after hatch year) birds. Posteruption feather samples were collected during the 1‐ to 3‐day field activities on the island (June 13–17, 2009, August 10–12, 2009, June 18–19, 2010, August 30, 2010, June 17–18, 2011, and August 15–17, 2011). Legacy samples (total *n *=* *7; three adults and four juveniles) consisted of feathers collected from two of the four active eyries or within the territories of known pairs from perches and plucking stations in 2006. Therefore, our legacy sample size represents between 25% (*n *=* *2/8) and 87.5% (*n *=* *7/8) of the peregrine falcons breeding in 2006. We assumed that molted feathers came from individuals that were residents of Kasatochi and not peregrines from nearby islands or nonbreeders, given their breeding site fidelity and territorial behavior (White, Clum, Cade, & Hunt, 2002). In addition, feather and egg shell membranes (*n *=* *31), collected as part of a larger regional study from peregrine falcons breeding throughout the Aleutian Islands, were included to derive insight into the contributions of islands in the reestablishment of falcons on Kasatochi Island (Figure 1).

### 2.2 Laboratory techniques

DNA was extracted using a salt extraction following Talbot et al. (2011). Genotype data were collected from 11 microsatellite loci (NVHfp5, 13‐1, 31, 46‐1, 54, 79‐4, 82‐2, 86‐2, 89‐2, 92, and 107; Nesje & Røed, 2000). This suite of microsatellite loci are sufficiently variable to have high confidence in our ability to identify unique individuals based on genotype data; probability of identity (PID) was 1.084e^−6^, and PID among first‐order relatives was 2.223e^−3^ within the Aleutian Islands (Talbot et al., 2011). Polymerase chain reaction (PCR) amplifications and thermocycler conditions followed Talbot et al. (2011). Samples were assayed soon after the completion of field activities each year. Feather samples were extracted separately from eggshell membrane samples. In addition, 35% of the samples were extracted, amplified and genotyped in duplicate for quality control. No inconsistencies in genotype scores were observed. Microsatellite genotype data are accessioned at the USGS Alaska Science Center data repository (http://dx.doi.org/10.5066/F7F18WV0).

### 2.3 Statistical analysis

Queller and Goodnight's (1989) index of relatedness (*rxy*) was calculated among pairs of peregrine falcons on Kasatochi Island within and across years and among individuals sampled throughout the archipelago, as well as averaged across all individuals within an island in a given year, using Identix 1.1 (Belkhir, Castric, & Bonhomme, 2002). Relatedness values range from −1 to 1, where *rxy* equals 0.5 for first‐order (i.e. full‐sibling, parent–offspring) relationships, 0.25 for second‐order (e.g., half‐sibling, grandparent) relationships, 0 for unrelated individuals, and −1 for outbred individuals. Isolation‐by‐distance (IBD) analyses were conducted to determine whether islands in closer geographic proximity were also more genetically similar (*F*_ST_) using Isolation by Distance web service version 3.23 (Bohonak, 2002) to further investigate the posteruption colonization of peregrine falcons to Kasatochi Island. Two IBD analyses were conducted: (1) among peregrine falcons sampled throughout the Aleutian Islands and the 2006 Kasatochi samples; and (2) among peregrine falcons sampled throughout the Aleutian Islands and the 2009–2011 Kasatochi samples. Geographic distances were calculated as straight‐line distance.

## 3 Results

### 3.1 Genetic relationship across years

Genotypes from peregrine falcons breeding on Kasatochi prior to the 2008 eruption did not share identical genotypes with any falcons sampled posteruption (2009–2011) and the proportion of familial relationships decreased each subsequent year (Table 1). However, DNA from a feather (Kas09‐003) collected in 2009 had high similarity with peregrine falcons sampled in 2006 (two comparisons with *rxy* > 0.5, a value expected between first‐order relatives, and four comparisons with *rxy* = 0.24–0.34, values expected between second‐order relatives). Among the posteruption samples, the genotype obtained from this same sample (Kas09‐003) was identical at all 11 loci to the genotype obtained from an eggshell membrane collected in 2010 (Kas10E02E). It is not possible that the feather and the egg shell membrane represent the same individual; rather this match likely represents a parent and its offspring, suggesting that at least one individual present on posteruption Kasatochi during 2009 returned the following year to Kasatochi Island to breed. Few first‐order familial relationships were observed between the remaining two comparisons (2009 & 2011, 2010 & 2011) with a greater proportion of second‐order relationships observed (Table 1).

**Table 1:** Percent pairwise relatedness (*rxy*) values within and among peregrine falcons sampled in 2006, and 2009–2011 on Kasatochi Island along with mean relatedness within years. Here, we define a first‐order familial relationship as having a *rxy* value greater than 0.40 (sharing at least one allele per locus) and second‐order relationship as having a *rxy* value between 0.20 and 0.39

	Familial relationship		*rxy* (variance)
	First order %	Second order %	
Kasatochi 2006 (*n *=* *7)	38.1 (*n *=* *8/21)	33.3 (*n *=* *7/21)	−0.240 (0.199)
& 2009	17.9 (*n *=* *5/28)	32.1 (*n *=* *9/28)	
& 2010	9.5 (*n *=* *4/42)	21.4 (*n *=* *9/42)	
& 2011	3.6 (*n *=* *1/28)	17.9 (*n *=* *5/28)	
Kasatochi 2009 (*n *=* *4)	16.7 (*n *=* *1/6)	0.0 (*n *=* *0/6)	−0.341 (0.102)
& 2010	20.8^a^ (*n *=* *5/24)	12.5 (*n *=* *3/24)	
& 2011	0.0 (*n *=* *0/16)	31.2 (*n *=* *5/16)	
Kasatochi 2010 (*n *=* *6)	6.7^b^ (*n *=* *1/15)	6.7 (*n *=* *1/15)	−0.179 (0.089)
& 2011	4.2 (*n *=* *1/24)	29.2 (*n *=* *7/24)	
Kasatochi 2011 (*n *=* *4)	33.3 (*n *=* *2/6)	33.3 (*n *=* *2/6)	−0.373 (0.259)

a Denotes a matching sample.

b Denotes egg shell membranes sampled from the same eyrie.

### 3.2 Genetic relationship within years

Overall *rxy* values estimated for Kasatochi peregrines within years were negative (Table 1). In general, *rxy* values were more negative than values estimated from other islands in the Aleutian archipelago (Table 2) and the Aleutian Island peregrine falcons as a whole (*rxy* = −0.010, variance = 0.066). Despite negative *rxy* values, greater than 66% of the comparisons indicated either a first‐ or second‐order familial relationship in 2006 and 2011 (Table 1). Fewer familial relationships were observed among 2009 and 2010 falcons.

**Table 2:** Percent pairwise relatedness (*rxy*) values among Kasatochi peregrine falcons sampled in 2006 and 2009–2011 with those peregrines sampled throughout the Aleutian chain. Here, we define a first‐order familial relationship as having a *rxy* value >0.40 (sharing at least one allele per locus) and second‐order relationship as having a *rxy* value between 0.20 and 0.39

	*rxy* (variance)	Familial relationships					
		Kasatochi 2006			Kasatochi 2009–2011		
		First order (%)	Second order (%)	Total (%)	First order (%)	Second order (%)	Total (%)
Amatignak	–	0.0 (*n *=* *0/7)	0.0 (*n *=* *0/7)	0.0	7.1 (*n *=* *1/14)	21.4 (*n *=* *3/14)	28.6
Amchitka	−0.068 (0.065)	8.8 (*n *=* *8/91)	14.3 (*n *=* *13/91)	23.1	7.1 (*n *=* *13/182)	13.2 (*n *=* *24/182)	20.3
Buldir	−0.271 (0.080)	0.0 (*n *=* *0/35)	25.7 (*n *=* *9/35)	25.7	10.0 (*n *=* *7/70)	4.3 (*n *=* *3/70)	14.3
Attu	−0.192 (0.393)	5.7 (*n *=* *2/35)	20.0 (*n *=* *7/35)	25.7	11.4 (*n *=* *8/70)	10.0 (*n *=* *7/70)	21.4
Commander Islands	−0.161 (0.064)	8.2 (*n *=* *4/49)	24.5 (*n *=* *11/49)	22.4	0.0 (*n *=* *0/98)	11.2 (*n *=* *11/98)	11.2

### 3.3 Genetic relationship throughout the Archipelago

Increasing genetic differentiation with increasing geographic distance was not observed within the Aleutian Archipelago pre‐eruption (*r *=* *.187, *p *=* *.28) or posteruption (*r *=* *.473, *p *=* *.14). However, the percentage of first‐ and second‐order familial relationships among Kasatochi Island and Amatignak Island (about 275 km west of Kasatochi; Figure 1) was higher in the posteruption samples than in the 2006 samples, although Amatignak was represented by a single feather sampled from an adult found dead in 2009 (Table 2). In contrast, the percentage of familial relationships between Kasatochi Island and the other sampled Aleutian Islands remained approximately similar, or lower, posteruption (Table 2).

## 4 Discussion

We found no genetic evidence to indicate that individual peregrine falcons known to have been present on Kasatochi Island pre‐eruption returned posteruption. However, we cannot rule out that pre‐eruption individuals returned; the absence of matching genotypes between pre‐eruption and posteruption individuals may be attributable to sampling bias. Although the number of occupied eyries on pre‐eruption Kasatochi is greater than found on average in the Aleutian Island archipelago (Ambrose et al., 1988), the four eyries occupied on Kasatochi in 2006 likely represented approximately eight adults, and the majority of the posteruption data for this study derived from feathers found during two 1‐ to 3‐day field activities on a small part of the island from 2009 to 2011. As well, peregrine falcons may demonstrate high territory turnover rates; peregrine falcons breeding on Haida Gwaii, British Columbia, another nonmigratory peregrine falcon population, have an adult median life expectancy of 2.8 years after their second year of life (Nelson, 1990). Nevertheless, our results did indicate that individuals closely related to peregrine falcons occupying pre‐eruption Kasatochi returned following the eruption. Fifty percent of comparisons indicated a familial relationship among peregrine falcons breeding in 2006 and those who left feathers in 2009 (Table 1). DNA from a feather collected in 2009 had high genetic affiliation with peregrine falcons sampled in 2006 and this sample matched at all 11 microsatellite loci with a sample collected in 2010. Although this match is between a feather (2009) and an egg shell membrane (2010) and, again, cannot represent the same individual, it does indicate a first‐order familial relationship (parent–offspring). Therefore, at least one peregrine, likely from a lineage that occupied Kasatochi prior to the eruption, was “observed” in 2009 and, in 2010, fledged the first documented avian young on Kasatochi Island posteruption. Thus, a genetic legacy of pre‐eruption falcons was present on posteruption Kasatochi Island via the presence of close relatives in 2009 and subsequent production of offspring in 2010.

**Figure 5:**
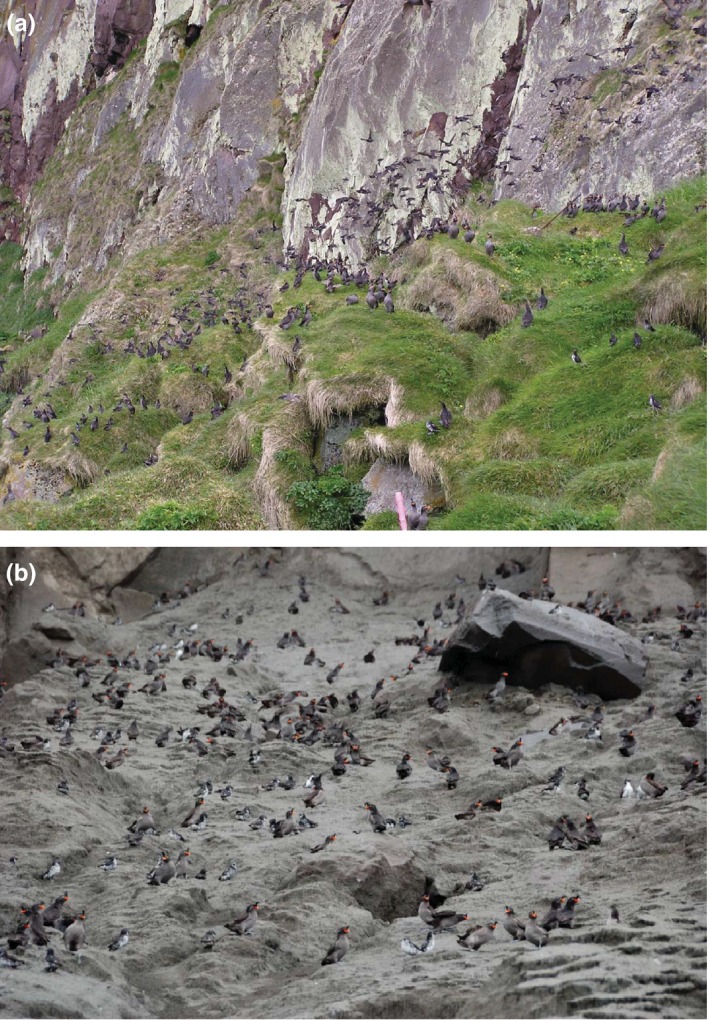
Auklets (*Aethia* sp.) on the colony surface at Kasatochi Island (a) before (June 2004; photograph by Brie Dummond) and (b) after the 2008 eruption (June 2009; photograph by Gary Drew). Photographs were taken from approximately the same location and scale

Peregrine falcons rapidly recolonized Kasatochi Island, with similar pre‐eruption numbers (feathers from four peregrine falcons were collected on Kasatochi in 2009, six in 2010, and four in 2011; Table 1; Figure 3). Behavioral characteristics of peregrine falcons breeding in the Aleutian Islands likely contributed to the rapid reestablishment as displaced falcons would have a propensity to return to Kasatochi (i.e., breeding site fidelity or philopatry) and displaced/neighboring falcons could easily return as they occupy nearby islands year‐round (White et al., 2002). This rapid reestablishment is particularly noteworthy when compared to other islands that either experienced a volcanic sterilization event or recently emerged. Peregrine falcons took more than 93 years (first record 1976; Rawlinson, Zann, van Balen, & Thornton, 1992) to successfully colonize Krakatau Islands after the eruption in 1883. On Anak Krakatau, a volcanic island that emerged from the sea in 1930, and later erupted in 1951–1952, peregrine falcons were not observed until almost 60 years later (1989; Zann & Darjono, 1992). Furthermore, peregrine falcons are only visitors to the volcanic islands of Motmot (emerged in 1968, Ball & Glucksman, 1975; Schipper, Shanahan, Cook, & Thornton, 2001) and Islas Revillagigedo (erupted in 1952; Hahn, Hogeback, Römer, & Vergara, 2012) and continue to be absent from Surtsey Island (emerged in 1963; Petersen, 2009). Although other volcanic islands have source populations in relatively close geographic proximity, peregrine falcons breeding in the Aleutian Islands are primarily nonmigratory with only some mid‐winter interisland movement and juvenile dispersal (White, 1975; White et al., 2002). The nearest nesting falcons are located on adjacent islands year‐round (ca. 19 km away), thereby increasing the opportunity for recolonization either via breeding or natal dispersal events. Colonization on other volcanic islands, notably Islas Revillagigedo and Surtsey, is restricted to migratory individuals, which may only pass by the islands infrequently, thereby reducing the likelihood of peregrine falcons becoming established on these islands. The pattern of posteruption succession of avian taxa observed on Kasatochi has been unique when compared to colonization patterns in the Krakataus, Islas Revillagigedo, and Surtsey. On volcanic islands for which peregrine falcons are not established, only larger bodied seabirds (*Phoebastria immutabilis*,* Phaethon aethereus*; Pitman & Balance, 2002) have colonized the islands in large numbers on San Benedicto, Islas Revillagigedo (where, historically, marine birds were abundant; Ball & Glucksman, 1975), and Surtsey (*Larus* sp.; Petersen, 2009). Only a few (<50) breeding birds (*Anas superciliosa* and *Hirundo tahitica*) are present on Motmot (Schipper et al., 2001). Among the Krakataus, community assembly was needed to facilitate the reintroduction of avian predators, such as the peregrine falcon. Zann and Darjono (1992) hypothesized that the abundance of small passerines enabled the oriental hobby (*F. severus*) to become established in 1985 on Anak Krakatau; the hobby was later displaced by peregrine falcon. Presumably on the Krakataus, the arrival of passerines was dependent on the presence of suitable vegetation and accompanying food source. In contrast, common avian prey species are ubiquitous (e.g., 5,000,000 least auklets, 1,000,000 crested auklets) throughout the Aleutian Islands (see Gibson & Byrd, 2007 for additional estimates). Extensive peregrine prey analyses from Amchitka Island, about 390 km west of Kasatochi (Figure 1), found six species, none of which breed on that island but occupy the surrounding waters, made up 69% of the prey (*n *=* *548 total prey items; White, Emison, & Williamson, 1973). Pre‐eruption densities of crested and least auklets in waters surrounding Kasatochi Island did not change posteruption (Drew, Dragoo, Renner, & Piatt, 2010), and lack of crevices and vegetation likely increased their susceptibility to predation by peregrine falcons (Figure 5; see figure 1 in Williams et al., 2010). Thus, the circumstances on Kasatochi with regard to this top avian predator support the heterotrophs‐first (Hodkinson, Webb, & Coulson, 2002; König, Kaufman, & Scheu, 2011) marine‐based model proposed for predaceous flies that rapidly colonized Kasatochi following the eruption (Sikes, O'Brien, and Baltesperger unpublished data, cited in Walker et al., 2013). We contend, therefore, that critical factors in the rapid reestablishment of Kasatochi Island, regardless of the genetic affinity of the recolonizing falcons, include the abundance of near‐shore and pelagic food sources through the Aleutians as well as on posteruption Kasatochi, coupled with high vagility of peregrine falcons. These characteristics would enhance rapid recolonization, bolstering contributions due to fidelity to site, whether natal or simply breeding site fidelity, and the species’ lack of dependence on vegetation for nesting.

Given our sample size, it is difficult to determine the specific origin(s) of all the falcons that colonized Kasatochi Island following the 2008 eruption, aside from the lineage considered to have derived from temporarily displaced Kasatochi peregrines. The correlation between genetic and geographic distance increased from the pre‐eruption to the posteruption time periods, albeit not significantly, is likely due to sample size limitations. These trends suggest early posteruption recruitment from displaced prior residents occupying nearby islands, augmented in subsequent years by immigrants from other islands. Dispersal propensity would likely be a beneficial evolutionary strategy for species occupying this highly dynamic landscape. Therefore, species, such as peregrine falcons, that have rapidly colonized (or recolonized) novel habitats in this region may be predisposed to exhibit a metapopulation dynamic and possess (or evolved) characteristics that exploit this interplay of source and sink dynamics among neighboring islands. Indeed, source sink dynamics have also been characterized for mainland peregrine falcons (e.g., Kauffman, Pollock, & Walton, 2004; Wootton & Bell, 2014). The Aleutian Island archipelago is a geologically dynamic region and geologic and modern records indicate high levels of volcanic activity (Jicha, Scholl, Singer, Yogodzinski, & Kay, 2006; Miller et al., 1998). Eruptions occur approximately every 21–80 years among volcanically active islands within the Andreanof Island group (Alaska Volcano Observatory 2013; Coats, 1950) and within the Archipelago, 14 of 52 historically active volcanoes have had a major eruption since 1990 (DeGange, 2010). The relatively frequent partial or full sterilization of islands along the Aleutian chain likely drives community dynamics within the archipelago, favoring species with high dispersal capability and colonization propensity, such as the peregrine falcon, and ultimately influencing species composition of re‐assembled communities. Given the influence of initial founder events on the success of subsequent colonization attempts by other species, this evolutionary strategy is likely reinforced through time via positive selection on characteristics that can exploit this ephemeral landscape.

## Conflict of Interest

The authors have no conflict of interest to declare.

## References

[ece32631-bib-0001] Alaska Volcano Observatory (2013). Retrieved from http://www.avo.alaska.edu/ Accessed 20 December 2013.

[ece32631-bib-0002] Ambrose, R. E. , Ritchie, R. J. , White, C. M. , Schempf, P. F. , Swem, T. , & Dittrick, R. (1988). Changes in the status of peregrine falcon populations in Alaska In Cade T. J., Enderson J. H., Thelander C. G. & White C. M. (Eds.), Peregrine falcon populations: Their management and recovery. Proceedings of the 1985 International Peregrine Conference (pp. 73–82). Boise, ID: The Peregrine Fund, Inc.

[ece32631-bib-0003] Ball, E. , & Glucksman, J. (1975). Biological colonization of Motmot, a recently‐created tropical island. Proceedings of the Royal Society of London B: Biological Sciences, 190, 421–442.

[ece32631-bib-0004] Belkhir, K. , Castric, V. , & Bonhomme, F. (2002). IDENTIX, a software to test for relatedness in a population using permutation methods. Molecular Ecology Notes, 2, 611–614.

[ece32631-bib-0005] Bohonak, A. J. (2002). IBD (Isolation by Distance): A program for analyses of isolation by distance. Journal of Heredity, 93, 153–154. 12140277 10.1093/jhered/93.2.153

[ece32631-bib-0006] Coats, R. R. (1950). Volcanic activity in the Aleutian Arc, U.S. Geological Survey Bulletin B 0974‐B.

[ece32631-bib-0007] DeGange, A. R. (2010) U.S. Geological Survey (USGS), Western Region, Kasatochi Volcano, Coastal and Ocean Science, U.S. Geological Survey Fact Sheet 2010‐3028.

[ece32631-bib-0008] DeGange, A. R. , Byrd, G. V. , Walker, L. R. , & Waythomas, C. F. (2010). Introduction – The impacts of the 2008 eruption of Kasatochi Volcano on terrestrial and marine ecosystems in the Aleutian Islands, Alaska. Arctic, Antarctic, and Alpine Research, 42, 245–249.

[ece32631-bib-0009] Drew, G. S. , Dragoo, D. E. , Renner, M. , & Piatt, J. F. (2010). At‐sea observations of marine birds and their habitats before and after the 2008 eruption of Kasatochi Volcano, Alaska. Arctic, Antarctic, and Alpine Research, 42, 325–334.

[ece32631-bib-0010] Drummond, B. A. , & Larned, A. L. (2007). Biological monitoring in the central Aleutian Islands, Alaska in 2007: Summary appendices. U.S. Fish and Wildlife Service Report, AMNWR 07/06, Homer, AK.

[ece32631-bib-0011] Fleischer, R. C. , McIntosh, C. E. , & Tarr, C. L. (1998). Evolution on a volcanic conveyor belt: Using phylogeographic reconstructions and K‐Ar based ages of the Hawaiian Islands to estimate molecular evolutionary rates. Molecular Ecology, 7, 533–545. 9628004 10.1046/j.1365-294x.1998.00364.x

[ece32631-bib-0012] Franklin, J. F. (2005). Reconfiguring disturbance, succession, and forest management: The science of Mount St. Helens In Dale V. H., Swanson F. J. & Crisafulli C. M. (Eds.), Ecological responses to the 1980 eruption of Mount St. Helens (pp. v–ix). New York: Springer.

[ece32631-bib-0013] Fridriksson, S. , & Magnússon, B. (1992). Development of the ecosystem on Surtsey with references to Anak Krakatau. GeoJournal, 28(2), 287–291.

[ece32631-bib-0014] Gibson, D. D. , & Byrd, G. V. (2007). Birds of the Aleutian Islands, Alaska, Series in Ornithology, No. 1. Cambridge, MA: Nuttall Ornithological Club and Washington, D.C.: American Ornithological Union.

[ece32631-bib-0015] Gudmundsson, F. (1966) Birds observed on Surtsey. Surtsey Research Progress Report II: 23–28.

[ece32631-bib-0016] Hahn, I. J. , Hogeback, S. , Römer, U. , & Vergara, P. M. (2012). Biodiversity and biogeography of birds in Pacific Mexico along an isolation gradient from mainland Chamela via coastal Marias to oceanic Revillagigedo Islands. Vertebrate Zoology, 62, 123–144.

[ece32631-bib-0017] Hodkinson, I. D. , Webb, N. R. , & Coulson, S. J. (2002). Primary community assembly on land – the missing stages: Why are the heterotrophic organisms always the first? Journal of Ecology, 90, 569–577.

[ece32631-bib-0018] Hoverman, J. T. , & Relyea, R. A. (2008). Temporal environmental variation and phenotypic plasticity: A mechanism underlying priority effects. Oikos, 117, 23–32.

[ece32631-bib-0019] Jicha, B. R. , Scholl, D. W. , Singer, B. S. , Yogodzinski, G. M. , & Kay, S. M. (2006). Revised age of Aleutian Island Arc formation implies high rate of magma production. Geology, 34, 661–664.

[ece32631-bib-0020] Kauffman, M. J. , Pollock, J. F. , & Walton, B. (2004). Spatial structure, dispersal, and management of a recovering raptor population. American Naturalist, 164, 582–597. 10.1086/424763 15540149

[ece32631-bib-0021] König, T. , Kaufman, R. , & Scheu, S. (2011). The formation of terrestrial food webs in glacier foreland: Evidence for the pivotal role of decomposer prey and intraguild predation. Pedologia, 54, 147–152.

[ece32631-bib-0022] Mazzola, M. B. , Chambers, J. C. , Blank, R. R. , Pyke, D. A. , Schupp, E. W. , Allcock, K. G. , ··· Nowak, R. S. (2011). Effects of resource availability and propagule supply on native species recruitment in sagebrush ecosystems invaded by *Bromus tectorum* . Biological Invasions, 13, 513–526.

[ece32631-bib-0023] Miller, T. P. , McGimsey, R. G. , Richter, D. H. , Riehle, J. R. , Nye, C. J. , Yount, M. E. , & Dumoulin, J. A. (1998) Catalog of the historically active volcanos of Alaska. U.S. Geological Survey Open‐File Report OF 98‐0582.

[ece32631-bib-0024] Nelson, R. W. (1990). Status of the peregrine falcon, *Falco peregrinus pealei*, on Langara Island, Queen Charlotte Islands, British Columbia (Canada). Canadian Field‐Naturalist, 109, 193–199.

[ece32631-bib-0025] Nesje, M. , & Røed, K. H. (2000). Microsatellite DNA markers from the gyrfalcon (*Falco rusticolus*) and their use in other raptor species. Molecular Ecology, 9, 1438–1440. 10972786 10.1046/j.1365-294x.2000.00999-4.x

[ece32631-bib-0026] Percy, D. M. , Garver, A. M. , Wagner, W. L. , James, H. F. , Cunningham, C. W. , Miller, S. E. , & Fleischer, R. C. (2008). Progressive island colonization and ancient origin of Hawaiian *Metrosideros* (Myrtaceae). Proceedings of the Royal Society of London B: Biological Sciences, 275, 1479–1490. 10.1098/rspb.2008.0191 PMC2602662 18426752

[ece32631-bib-0027] Petersen, A. (2009). Formation of a bird community on a new island, Surtsey, Iceland. Surtsey Research, 12, 133–148.

[ece32631-bib-0028] Pitman, R. L. , & Balance, L. T. (2002). The changing status of marine birds breeding at San Benedicto Island, Mexico. Wilson Bulletin, 114, 11–19.

[ece32631-bib-0029] Queller, D. C. , & Goodnight, K. F. (1989). Estimating relatedness using genetic markers. Evolution, 43, 258–275. 10.1111/j.1558-5646.1989.tb04226.x 28568555

[ece32631-bib-0030] Rawlinson, P. A. , Zann, R. A. , van Balen, S. , & Thornton, I. W. B. (1992). Colonization of the Krakatau Islands by vertebrates. GeoJournal, 28(2), 225–231. 10.1073/pnas.85.2.515 PMC279581 3422440

[ece32631-bib-0031] Ricklefs, R. E. (2010). Dynamics of colonization and extinction on islands: Insights from lesser Antillean birds In Losos J. B. & Ricklefs R. E. (Eds.), The theory of island biogeography revisited (pp. 338–414). New Jersey: Princeton University Press.

[ece32631-bib-0032] Schipper, R. D. , Shanahan, M. , Cook, S. , & Thornton, I. W. B. (2001). Colonization of an island volcano, Long Island, Papua New Guinea, and an emergent island, Motmot, in its caldera lake. III Colonization by birds. Journal of Biogeography, 28, 1339–1352.

[ece32631-bib-0033] Shaw, K. L. (1996). Sequential radiations and patterns of speciation in the Hawaiian cricket genus *Laupala* inferred from DNA sequences. Evolution, 50, 237–255. 10.1111/j.1558-5646.1996.tb04488.x 28568854

[ece32631-bib-0034] Talbot, S. L. , Palmer, A. , Sage, G. K. , Sonsthagen, S. , Swem, T. , Brimm, D. , & White, C. M. (2011). Lack of genetic polymorphism among Peregrine Falcons of Fiji. Journal of Avian Biology, 42, 415–428.

[ece32631-bib-0035] Thornton, I. W. B. (1984). Krakatau – The development and repair of a tropical ecosystem. Ambio, 13, 217–225.

[ece32631-bib-0036] Thornton, I. W. B. , Zann, R. A. , & Stephenson, D. G. (1990). Colonization of the Krakatau islands by land birds, and the approach to an equilibrium number of species. Philosophical Transactions of the Royal Society of London B, 328, 55–93.

[ece32631-bib-0037] Walker, L. R. , Sikes, D. S. , DeGange, A. R. , Jewett, S. C. , Michaelson, G. , Talbot, S. L. , … Williams, J. C. (2013). Biological legacies: Direct early ecosystem recovery and food web reorganization after a volcanic eruption in Alaska. Ecoscience, 20, 1–12.

[ece32631-bib-0038] Waters, J. M. , Fraser, C. I. , & Hewitt, G. M. (2012). Founder takes all: Density‐dependent processes structure biodiversity. Trends in Ecology and Evolution, 28, 78–85. 23000431 10.1016/j.tree.2012.08.024

[ece32631-bib-0039] White, C. M. (1975) Peregrine falcon in the Aleutian Islands In Murphy J. R., White C. M. & Harrell B. E. (Eds.), Population status of raptors: Proceedings of the Conference on Raptor Conservation Techniques 6:33–50.

[ece32631-bib-0040] White, C. M. (1976). Aleutian Islands In Fyfe R., Temple S. A. & Cade T. J. (Eds.), The 1975 North American Peregrine falcon survey. Canadian Field‐Naturalist vol 90.

[ece32631-bib-0041] White, C. M. , Clum, N. J. , Cade, T. J. , & Hunt, W. G. (2002). Peregrine Falcon (*Falco peregrinus*) In Poole A. & Gill F. (Eds.), The Birds of North America, No. 660. Ithaca: Cornell Lab of Ornithology Retrieved from https://birdsna.org/Species-Account/bna/species/perfal. doi: 10.2173/bna.660

[ece32631-bib-0042] White, C. M. , Emison, W. B. , & Williamson, F. S. L. (1971). Dynamics of raptor populations on Amchitka Island, Alaska. BioScience, 21, 622–627.

[ece32631-bib-0043] White, C. M. , Emison, W. B. , & Williamson, F. S. L. (1973). DDE in a resident Aleutian Island peregrine population. Condor, 75, 306–311.

[ece32631-bib-0044] Williams, J. C. , Drummond, B. A. , & Buxton, R. T. (2010). Initial effects of the August 2008 volcanic eruption on breeding birds and marine mammals at Kasatochi Island, Alaska. Arctic, Antarctic, and Alpine Research, 42, 306–314.

[ece32631-bib-0045] Wootton, J. T. , & Bell, D. A. (2014). Assessing predictions of population viability analysis: Peregrine Falcon populations in California. Ecological Applications, 24, 1251–1257. 10.1890/13-1323.1 29160650

[ece32631-bib-0046] Yang, S. , Bishop, J. G. , & Webster, M. S. (2008). Colonization genetics of an animal‐dispersed plant (*Vaccinium membranaceum*) at Mount St Helens, Washington. Molecular Ecology, 17, 731–740. 18194163 10.1111/j.1365-294X.2007.03625.x

[ece32631-bib-0047] Zann, R. A. , & Darjono, Drs. (1992). The birds of Anak Krakatau: The assembly of an avian community. GeoJournal, 28, 261–270.

